# Life-threatening hemobilia caused by hepatic pseudoaneurysm after T-tube choledochostomy: report of a case

**DOI:** 10.1186/1471-230X-10-81

**Published:** 2010-07-14

**Authors:** Yueh-Tsung Lee, Ho Lin, Kuan-Yung Chen, Hurng-Sheng Wu, Min-Ho Hwang, Sheng-Lei Yan

**Affiliations:** 1 Department of Surgery, Chang-Bing Show Chwan Memorial Hospital, Changhua County, Taiwan, ROC; 2 Department of Life Sciences, National Chung-Hsing University, Taichung City, Taiwan, ROC; 3 Department of Radiology, Chang-Bing Show Chwan Memorial Hospital, Changhua County, Taiwan; 4 Division of Gastroenterology, Department of Internal Medicine, Chang-Bing Show Chwan Memorial Hospital, Taiwan, ROC

## Abstract

**Background:**

Hemobilia is a rare but lethal biliary tract complication. There are several causes of hemobilia which might be classified as traumatic or nontraumatic. Hemobilia caused by pseudoaneurysm might result from hepatobiliary surgery or percutaneous interventional hepatobiliary procedures. However, to our knowledge, there are no previous reports pertaining to hemobilia caused by hepatic pseudoaneurysm after T-tube choledochostomy.

**Case presentation:**

A 65-year-old male was admitted to our hospital because of acute calculous cholecystitis and cholangitis. He underwent cholecystectomy, choledocholithotomy via a right upper quadrant laparotomy and a temporary T-tube choledochostomy was created. However, on the 19th day after operation, he suffered from sudden onset of hematemesis and massive fresh blood drainage from the T-tube choledochostomy. Imaging studies confirmed the diagnosis of pseudoaneurysm associated hemobilia. The probable association of T-tube choledochostomy with pseudoaneurysm and hemobilia is also demonstrated. He underwent emergent selective microcoils emobolization to occlude the feeding artery of the pseudoaneurysm.

**Conclusions:**

Pseudoaneurysm associated hemobilia may occur after T-tube choledochostomy. This case also highlights the importance that hemobilia should be highly suspected in a patient presenting with jaundice, right upper quadrant abdominal pain and upper gastrointestinal bleeding after liver or biliary surgery.

## Background

Hemobilia is a rare but lethal biliary tract complication [1]. Patients with hemobilia usually present with right upper quadrant abdominal pain, jaundice and upper gastrointestinal (UGI) bleeding [2]. There are several causes of hemobilia which might be classified as traumatic [3-12] or nontraumatic [12-19]. Hemobilia caused by pseudoaneurysm might be due to abdominal trauma [2], liver and biliary surgery [8,9,11], percutaneous interventional hepatobiliary procedures [7,10,12,20,21], tumor [16-19,22] or other infectious processes such as liver abscess [13], cholecystitis [23], cholangitis [17], and pancreatitis [5,12,17]. To our knowledge, there are no previous reports pertaining to hemobilia caused by hepatic pseudoaneurysm after T-tube choledochostomy. We describe here a novel case of hemobilia caused by pseudoaneurysm in the proper hepatic artery after T-tube choledochostomy. The probable association of T-tube choledochostomy with pseudoaneurysm and hemobilia is discussed.

## Case Presentation

A 65-year-old male was admitted to our hospital because of right upper quadrant abdominal pain, jaundice and fever for 2 days. He denied history of any systemic disease. He had no history of previous abdominal surgery. At initial presentation, his body temperature was 38.6°C, blood pressure was 142/82 mmHg, pulse rate was 92/min, and respiratory rate was 22/min. Physical examination revealed jaundice, and tenderness with light palpation at the right upper quadrant of abdomen. Murphy's sign was positive. There was no palpable mass and Courvoisier's sign was negative. The bowel sound was normal in peristalsis and no abdominal bruit was found on auscultation.

Laboratory studies demonstrated a white blood count of 10,800/mm^3 ^(normal, 4,500 to 10,000/mm^3^), a hemoglobin level of 15.3 g/dL (normal 14-18 g/dL), and a platelet count of 22.3 × 10^4 ^/mm^3 ^(normal, 13-40 × 10^4 ^/mm^3^). The liver profile showed total bilirubin = 4.3 mg/dL (normal, 0.2-1.2 mg/dL), direct bilirubin = 3.2 mg/dL (normal, 0-0.4 mg/dL), alanine aminotransferase = 108 IU/L (normal, 4-44 IU/L), aspartate aminotransferase = 103 IU/L (normal, 8-38 IU/L), and alkaline phosphatatse = 327 IU/L (normal, 104-338IU/L). The serum levels of amylase and lipase were 166 U/L (normal, 43-116 U/L) and 208 U/L (normal, 13-60 U/L) respectively. Blood cultures were negative for aerobic and anaerobic organisms. Abdominal ultrasonography revealed a distended gallbladder with thickened wall and filled with stones. A small amount of ascites was detected in the Morison's pouch. The common bile duct (CBD) was mildly dilated with some hyperechoic spots, indicating sandy CBD stones.

Because acute calculous cholecystitis and cholangitis were impressed on clinical grounds, he underwent cholecystectomy and choledocholithotomy via a right upper quadrant laparotomy. At operation, an inflamed gallbladder with fibrin coating on the edematous wall was found. The Calot's triangle showed dense adhesion and the CBD was distended. The CBD was explored to remove sandy stones, debris and sticky bile. A temporary T-tube choledochostomy was created and Penrose drains were set in the Morison's pouch.

**Figure 1:**
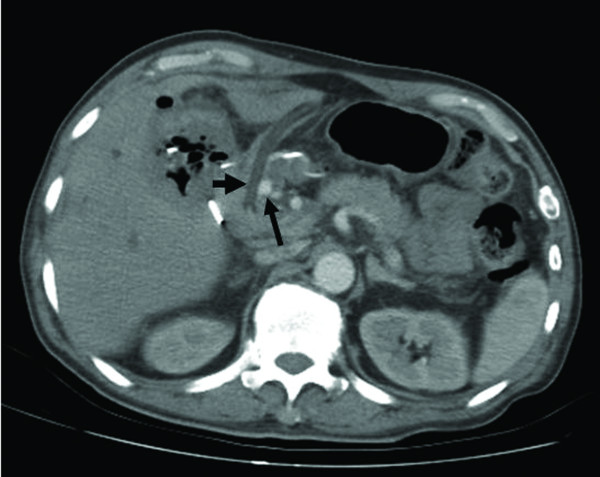
**Contrasted enhanced CT of the abdomen was arranged showing a round and hyperdense lesion (long arrow) abutting the T-tube choledochostomy (short arrow)**.

**Figure 2:**
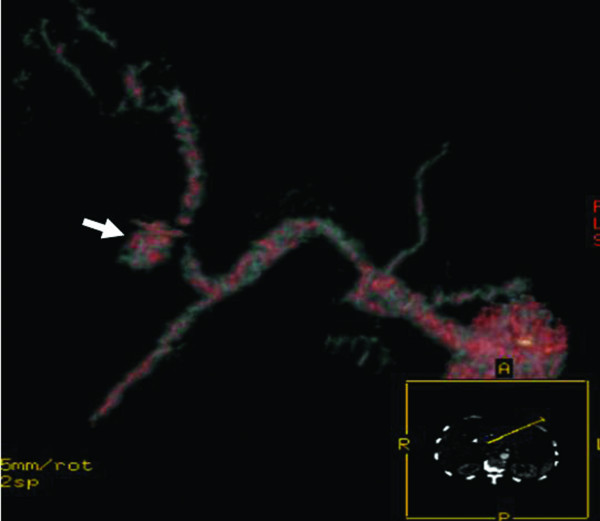
**Reconstructive CT image showing a saccular mass protruding from the proper hepatic artery**.

**Figure 3:**
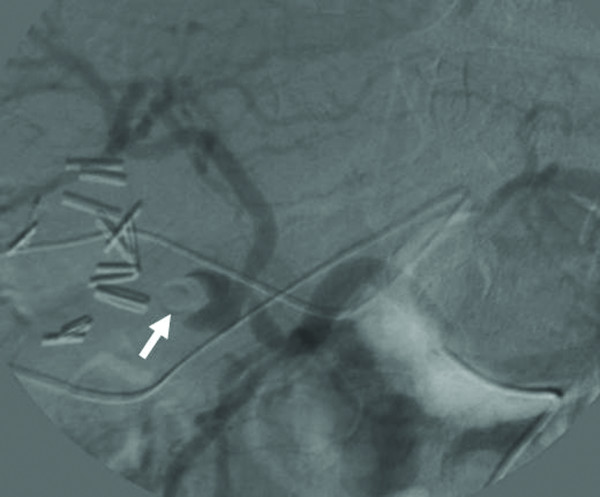
**Transarterial angiographic view showing a contrast-filled lesion arising from proper hepatic artery**. Adjacent to the contrast-filled lesion was the T-tube (arrow).

**Figure 4:**
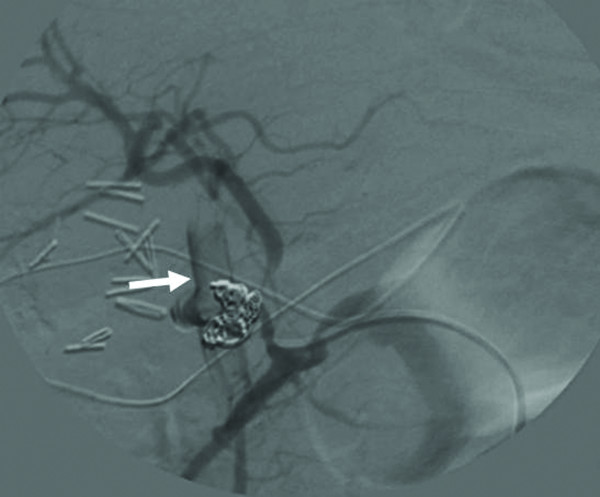
**Transarterial angiographic view showing that contrast drained into the T-tube choledochostomy (arrow)**.

**Figure 5:**
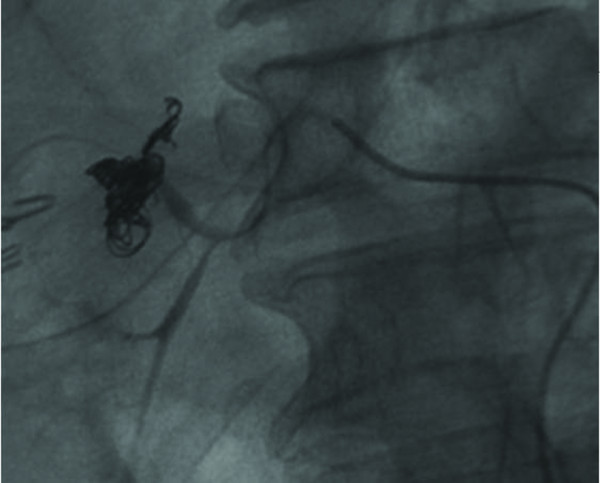
**Transarterial angiographic view showing that the pseudoaneurym was occluded using selective microcoils emobolization**.

The patient recovered well after the operation without fever episodes or jaundice. He was discharged on the 14^th ^postoperative days. However, on the 19^th ^day after operation, he suffered from sudden onset of hematemesis and massive fresh blood drainage from the T-tube choledochostomy. Hypovolemic shock developed thereafter. He was sent to emergent department of our institution. Laboratory examination revealed a decreased hemoglobin level of 7.0 g/dL and an increased total bilirubin level of 2.1 mg/dL. Because hemobilia was highly suspected a contrast enhanced computed tomography (CT) of the abdomen was arranged emergently, revealing a round and hyperdense lesion abutting the T-tube choledochostomy (Figure 1). The reconstructive CT image further demonstrated a saccular mass protruding from the proper hepatic artery (Figure 2). The angiography disclosed a contrast-filled lesion arising from proper hepatic artery, which suggested a pseudoaneurysm formation (Figure 3). The contrast drained into the T-tube choledochostomy directly (Figure 4), indicating that pseudoaneurysm was the cause of hemobilia in our patient. He underwent emergent selective microcoils embolization to occlude the feeding artery of the pseudoaneurysm and the bleeding ceased thereafter (Figure 5). He recovered uneventfully with normal liver profile after a three-month period of follow up.

## Conclusions

Hemobilia, a rare but lethal disaster, was first described by Sandblom in 1948 as the situation of bleeding into the biliary tract following trauma [1]. The classical presentation of hemobilia, also known as Quinckes' triad, includes right upper quadrant abdominal pain, UGI bleeding and jaundice [2]. Blunt and penetrating abdominal trauma, accounting for half of the cases, are the leading causes of hemobilia [2]. Liver and biliary surgery [4,8,9,11], percutaneous liver biopsy [4,12], percutaneous liver tumor ablation [21], and percutaneous transhepatic biliary drainage and stent placement [4,7,10,20] contribute to the other causes of traumatic hemobilia. Nontraumatic causes of hemobilia include aneurysm or pseudoaneurysm caused by liver abscess [13], choledocholithiasis and related infection [14,23], pancreatitis [5,12,15,16,18], and tumor [16-19,22]. However, to the best of our knowledge, there are no previous reports in the English literature regarding hepatic pseudoaneurysm associated hemobilia after T-tube choledochostomy. This unusual case could be added to the causal list of hemobilia after surgery.

As for the etiological cause of pseudoaneurysm formation in our case, we propose that mechanical compression of the T-tube on the bile duct mucosa and adjacent proper hepatic artery might have caused erosion of the blood vessel, and induced pseudoaneurysm formation. There are evidences that might support our proposal. Firstly, anatomically speaking, the common bile duct runs along with the proper hepatic artery within the hepatoduodenal ligament. Secondly, the contrast-filled hyperdense pseudoaneurysm found at abdominal CT was just close to the T-tube. The angiographic features further disclosed that the lunar pseudoaneurysm was compressed by the T-tube and the contrast flew directly from the proper hepatic artery into the T-tube.

The diagnosis of hemobilia can be confirmed by endoscopy examination or endoscopic retrograde cholangiopancreatography to directly observe bleeding from the ampulla of Vater [12]. Doppler ultrasound can be used to detect the pulsatile mass with blood flow into the biliary tracts [24]. Tumors, abscesses, and the location of aneurysms and pseudoaneurysms can be identified by abdominal CT [25]. Furthermore, contrast-enhanced magnetic resonance imaging can be used to demonstrate the correlation of blood flow with bile ducts to support the diagnosis of hemobilia [26]. However, transarterial angiography should be considered as the imaging modality of choice, because it can be used to detect and embolize the bleeders [2,9,22,27,28]. Surgical ligation, excision of aneurysm and pseudoaneurysm or partial hepatectomy is only conserved for failed embolization or tumor-related hemobilia [2,22]. Furthermore, recent articles suggest that minimally invasive procedures were offered for treatment of hemobilia, especially for tumor associated hemobilia [18,19,22].

In conclusion, pseudoaneurym associated hemobilia may occur after T-tube choledochostomy. This case also highlights the importance that hemobilia should be highly suspected in a patient presenting with jaundice, right upper quadrant abdominal pain and UGI bleeding after liver and biliary surgery. Once a diagnosis is established, angiographic intervention should be performed without delay because it is a life-threatening situation.

## Consent

Written informed consent was obtained from the patient for publication of this case report and any accompanying images. A copy of the written consent is available for review by the Editor-in-Chief of this journal.

## Abbreviations

UGI: upper gastrointestinal; CT: computed tomography; CBD: common bile duct.

## Competing interests

The authors declare that they have no competing interests.

## Authors' contributions

All authors have read and approved the final version of manuscript. LYT: surgical operation, drafting the manuscript; LH: helped to draft and edit the manuscript; CKY: carried out radiological intervention; WHS: involved in the clinical management of the patient; HMH: revised the manuscript critically and added substantial intellectual content; YSL: final review, supervision of scientific content of manuscript.

## Pre-publication history

The pre-publication history for this paper can be accessed here:

http://www.biomedcentral.com/1471-230X/10/81/prepub
